# Electrofusion of single cells in picoliter droplets

**DOI:** 10.1038/s41598-018-21993-8

**Published:** 2018-02-27

**Authors:** Rogier M. Schoeman, Wesley T. E. van den Beld, Evelien W. M. Kemna, Floor Wolbers, Jan C. T. Eijkel, Albert van den Berg

**Affiliations:** 1 Colorado School of Mines, Chemical and Biological Engineering Department, Golden, 80401 Colorado, United States; 2 University of Twente, MESA+ Institute for Nanotechnology and MIRA Institute for Biomedical Technology, Faculty of Electrical Engineering, Mathematics and Computer Science, BIOS Lab on a Chip group, 7500AE Enschede, The Netherlands; 3 Saxion University of Applied Sciences, Life Science Engineering and Design, PO Box 70.000, 7500KB Enschede, The Netherlands

## Abstract

We present a microfluidic chip that enables electrofusion of cells in microdroplets, with exchange of nuclear components. It is shown, to our knowledge for the first time, electrofusion of two HL60 cells, inside a microdroplet. This is the crucial intermediate step for controlled hybridoma formation where a B cell is electrofused with a myeloma cell. We use a microfluidic device consisting of a microchannel structure in PDMS bonded to a glass substrate through which droplets with two differently stained HL60 cells are transported. An array of six recessed platinum electrode pairs is used for electrofusion. When applying six voltage pulses of 2–3 V, the membrane electrical field is about 1 MV/cm for 1 ms. This results in electrofusion of these cells with a fusion yield of around 5%. The operation with individual cell pairs, the appreciable efficiency and the potential to operate in high-throughput (up to 500 cells sec^−1^) makes the microdroplet fusion technology a promising platform for cell electrofusion, which has the potential to compete with the conventional methods. Besides, this platform is not restricted to cell fusion but is also applicable to various other cell-based assays such as single cell analysis and differentiation assays.

## Introduction

Currently, the demand for antibodies is increasing every year. They are used for research, as well as for diagnostic and therapeutic purposes^1,2^ such as to treat different types of cancer, Alzheimer’s disease, Ebola, rheumatoid arthritis and multiple sclerosis^3–6^. For all of these applications, very promising results have been obtained. The production of antibodies primarily relies on the creation of antibody-secreting hybridomas^2,7^ that are obtained via cell fusion of antibody-producing B cells and immortal myeloma cells. B cells have a short lifespan *in vitro* and hence produce antibodies only briefly. Myeloma cells on the other hand proliferate rapidly *in vitro* due to their cancerous characteristics. By fusing these two cells, a hybrid cell (hybridoma) can be formed, capable of producing antibodies and able to proliferate rapidly *in vitro*, securing the prolonged production of antibodies.

Complete cell fusion occurs by a sequence of outer membrane fusion and nuclear fusion. For fusion of the outer membranes, the membranes of the two cells have to be brought in close contact and subsequently be subjected to a fusion stimulus. The newly formed fused cell then still contains two separate nuclei, and in a second step nuclear fusion has to follow. While membrane fusion can be induced by several methods, unfortunately nuclear fusion is a random process which can hardly be influenced. Nuclear fusion usually takes place within 1–2 weeks after the membrane fusion. The newly formed cell has to recombine the two sets of DNA, present in the two nuclei, which results in a hybridoma which contains genetic material from both parental cells and will display a mixture of the characteristics from both cells^8,9^.

Current methods for hybridoma formation rely on random cell pairing in large fusion vessels^10^, resulting in both a low fusion efficiency, ranging from 0.06–0.24% and a functional hybridoma generation efficiency ranging from 0.002–0.05%^11,12^. Although higher cell fusion efficiencies ranging from 8.4–64% are reported, they remain unclear about the functional hybridoma generation efficiency^13–18^. Moreover the above mentioned percentages include multiple (more than two cells) cell fusion events^13–16^. Also the cells are aligned randomly by dielectrophoresis (DEP) reducing the hybridoma generation yield^18^. Even though this makes the currently used cell fusion methods expensive, time consuming and very inefficient, cell fusion still plays a central role in biotechnology, with a crucial role in the generation of monoclonal antibody-secreting hybridomas^11,19–25^ and furthermore applications such as the determination of the genetic make-up of organisms and cloning of mammals^26^. Here we present a new, low cost, cell fusion method that is able to fuse cells with similar efficiency. However, this method has the potential of becoming high-throughput electrofusion platform.

To get the two different cell types in close proximity, we bring them in the confined space of a microdroplet and electrofuse them. This arrangement provides a much better control of electroporation process. For this purpose we use of a microfluidic droplet platform combining several functionalities that were developed in previous work. Droplet-based microfluidics for biological experiments has received increasing interest in recent years, for several reasons^12,27^. Firstly, it enables the generation of monodisperse, compartmentalized microreaction vessels at high frequencies (kHz). Secondly, the encapsulation possibilities are enormous, ranging from bacteria to multicellular organisms^28^, enabling a wide range of biological assays that can be performed in a high-throughput manner. Thirdly, the droplets can be manipulated by (electro)coalescence, splitting and sorting^29–31^. Our final aim is to develop a high-throughput microfluidic system to create functional hybridomas with appreciable efficiency for antibody production in a controlled fashion^32^. Our droplet platform enables hybridoma generation and has the potential of becoming a continuous, high-throughput method.

**Figure 1:**
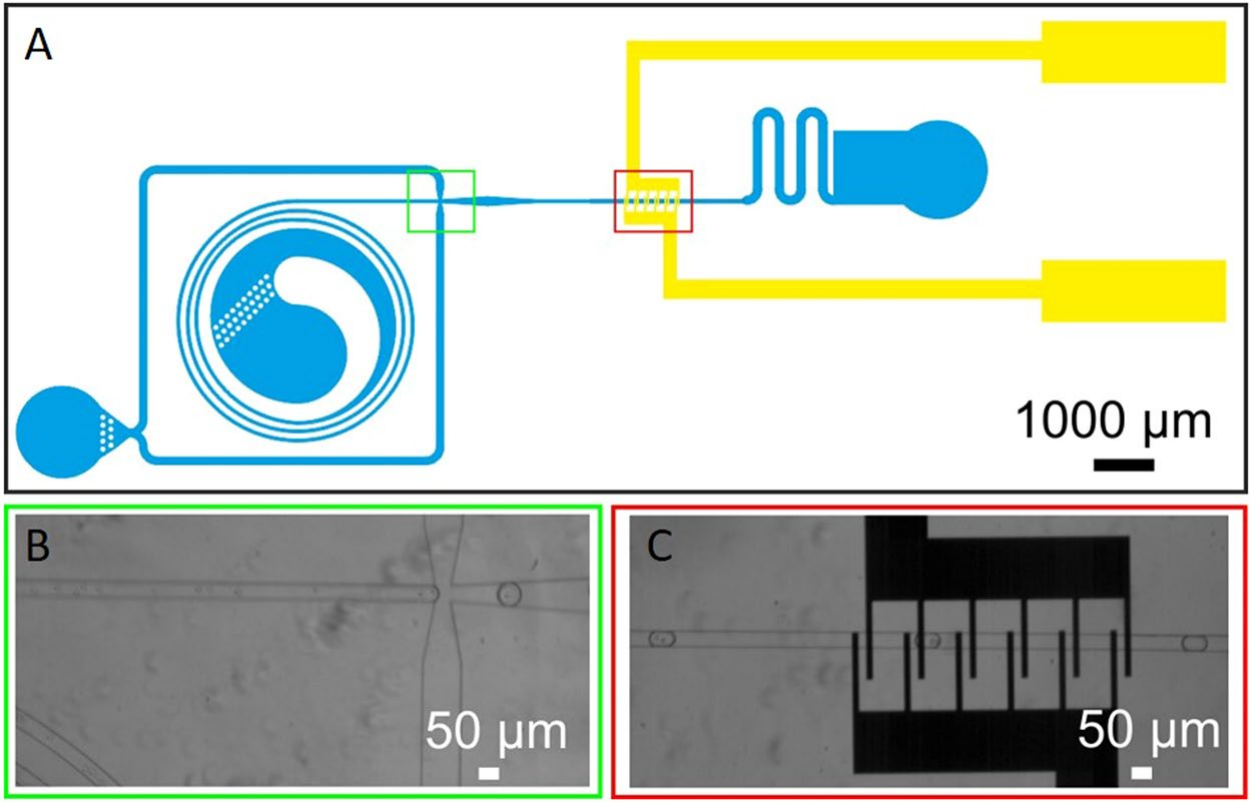
Microfluidic platform for cell electrofusion in droplets. (**A**) A schematic overview is shown with a yin-yang shaped cell solution inlet that functions as an elongated funnel to gradually introduce the cells into the channel to prevent the cells from clogging up the channel, and colored inserts of the different functionalities of the chip. (**B**) In green, cell encapsulation in droplets. (**C**) In red, droplets containing two cells passing the electrode array, consisting of six electrode pairs, all capable of giving a pulse of certain preset strength.

The platform discussed in this paper consists of two essential elements, i.e., for encapsulation of cells in droplets and for cell electrofusion in the droplets (Fig. 1). From the wide range of available components described in literature^33–47^, we have selected the appropriate ones and modified and optimized their design for integration in our electrofusion platform, in order to assess the feasibility of electrofusion of cells in a droplet^32,33^. For electrofusion, cell-containing droplets are transported over a sequence of six electrode pairs. To gain insight in the transient changes in the electrical potential distribution inside a cell-loaded droplet when the droplet moves over an electrode pair, the electrical potential distribution was calculated using an equivalent electrical circuit model. The calculated values were used to interpret the electroporation experiments performed^48–50^. To demonstrate the process and investigate the procedure, droplets containing two differently stained HL60 cells were generated. Due to the different fluorescent staining, the process of electrofusion could be assessed in detail at the single cell level. As we will show in the results section, we demonstrate for the first time successful electrofusion of cells in droplets. Moreover, the presented platform is not restricted to cell fusion and is applicable for various other cell-based assays such as single cell analysis and differentiation assays.

## Results

### Modeling of the electrical field

**Figure 2:**
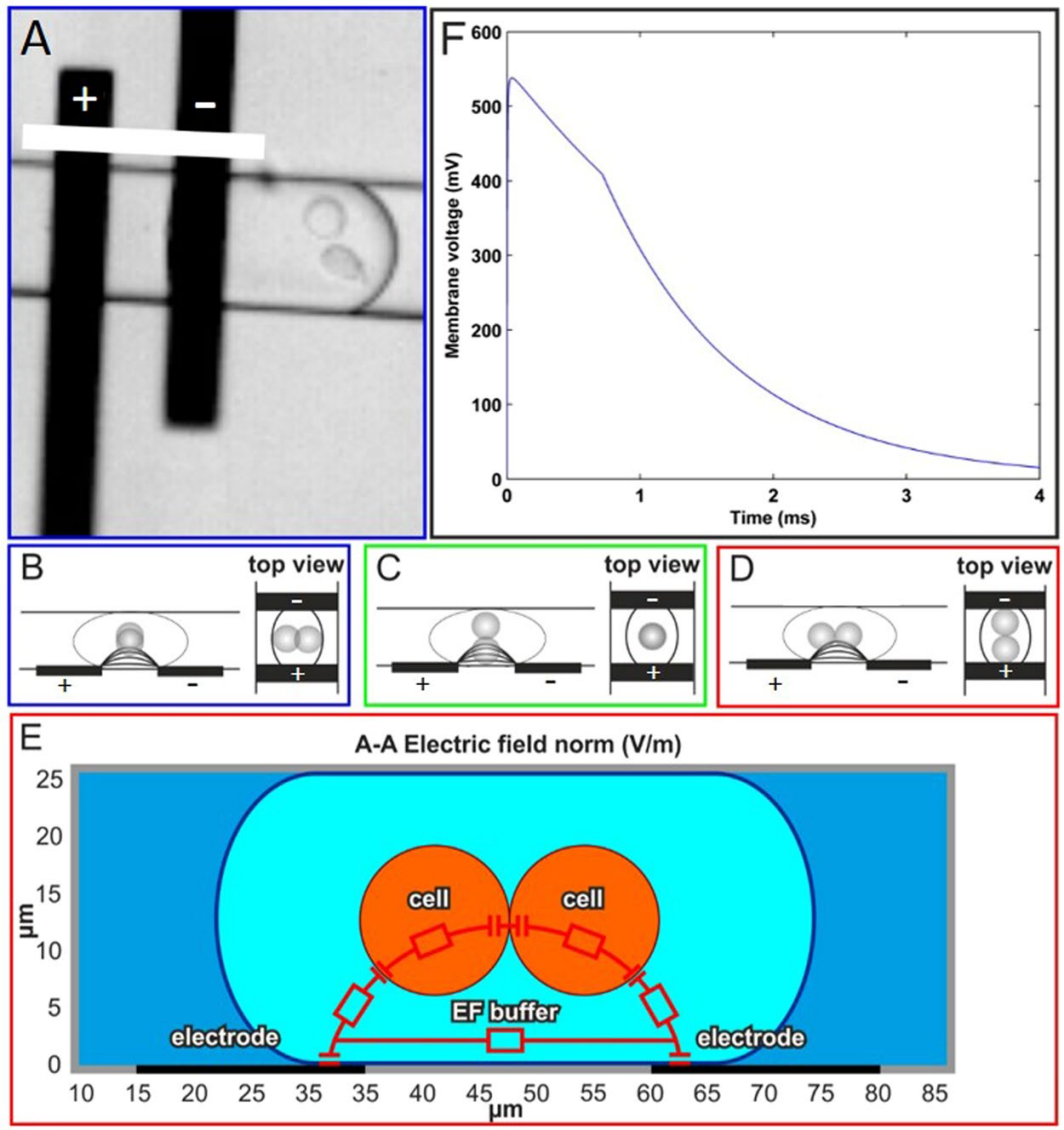
Simulation of electric field in a droplet. (**A**) A snapshot from a high-speed recording, reflecting situation in (Fig. 2B). The white bar +  indicates the plane in which the field lines of the electric field between the electrodes propagate. (**B**–**D**) Schematic representations of different situations in which the cells can align in a droplet, of which (**D**) is the ideal situation. (**E**) Cell membrane voltage model of the electric field in a droplet passing one electrode pair. In this side view, one of the electrodes has a voltage of 3 V and the other is grounded. Both electrodes are placed at the bottom. (**E**) The induced electric field in the membrane is strong enough (1 MV/cm) to result in electrofusion.

In order to successfully realize electrofusion of cells inside a droplet, a sufficiently high voltage across the cell membranes of the paired cells is essential. Therefore, the transient changes in the electrical potential inside a droplet were calculated using an equivalent electrical circuit model. Since planar electrodes are used, the cells will often not have an optimal orientation with respect to the applied electrical field (Fig. 2A–C). The ideal cell orientation for electrofusion is when the plane of the contacting cell membranes lies perpendicular to the electric field (Fig. 2D). This best-case situation was considered for the electrical circuit model (Fig. 2E).

The numerically approximated potential over a single cell membrane in time is shown in Fig. 2F, where t = 0 is the moment the droplet has reached the second electrode. The time constant to charge the double layer at the electrode interface is found to be about 4 ms and the time constant for charging the cell membrane about 1 µs. Thus the cell membrane is charged almost instantaneously with respect to the double layer membrane. The time both electrodes are in contact with the droplet at the experimental droplet velocity is 0.71 ms. During this time it can be seen the membrane voltage decays from 550 to 400 mV. When the second electrode is fully covered by the droplet, the membrane voltage starts to drop exponentially. These voltages are equivalent to an electric field in the cell membrane of 1 MV/cm, which is sufficient for membrane electroporation^51,52^ and hence can enable subsequent electrofusion of the two encapsulated cells^53,54^ The results from this model are in accordance with the results from the electroporation experiments, where the application of 6 pulses with the previous parameters showed successful electroporation. Moreover from our model the current in our system is roughly 0.1 uA for about 2 ms, resulting in an energy consumption of approximately 0.6 nJ. Assuming no cooling from the side walls, no liquid flow and considering the volume of electrofusion buffer close to one pair of electrodes (0.125 nL), the liquid could heat up about 1–2 °C. Thus in the worst case scenario after an array of 6 electrode pairs, the solution could heat up about 6–12 °C. The experiments were carried out at room temperature and therefore the overal temperture of the droplets and the cells could never surpass 37 ^o^C. Also to establish viability, cells were stained with calcein AM before the experiments. Calcein AM is a green cell dye that is only visible if the cells are alive. 95% of the cells were positive (green) after the experiment with or without applying the fusion voltage.

### Experimental procedure and setup

To generate the droplets for the cell electrofusion experiments, we used a flow-focusing geometry^33^. The cell volume fraction was 1.0%, resulting in 15% of the droplets containing two HL60 cells. HL60 cells were used because they are suspension cells and have dimensions similar to B cells and myeloma cells. Moreover, they consist almost completely out of a nucleus which facilitates visualization of electrofusion with nuclear cell staining. Finally, HL60 cells are easier to maintain in a cell culture. The settings of the performed electrofusion experiments were an applied voltage of 3 V and a droplet velocity of 28 mm/s.

Besides the magnitude of the electric field, as described above, various other factors play a role for successful electrofusion to occur. A crucial factor is the droplet traveling speed. As seen in the preceding section, the droplet speed and the electrode width determine the length of the applied fusion pulse. To increase the pulse length, the droplet speed can be decreased by reducing the net flow rate or widening the channel to slow down the droplet. To reduce the pulse length, the droplet speed can be increased by increasing the net flow rate. However, to generate droplets that are as small and stable as possible, the flow rate range is set^32^. The preferred droplet volume is ~18 pL (defined by the diameter of two encapsulated HL60 cells of d = 13 µm) while in practice at the optimal flow rate for droplet formation, the droplets have a volume of 35 pL. The electrode width can also be adjusted, but should not be more than the droplet length, as the droplet must be in contact with both electrodes to create the electric field over the encapsulated cells. Finally, since previous results showed that applying multiple pulses resulted in higher fusion efficiencies^55^, multiple electrode pairs were employed.

**Figure 3:**
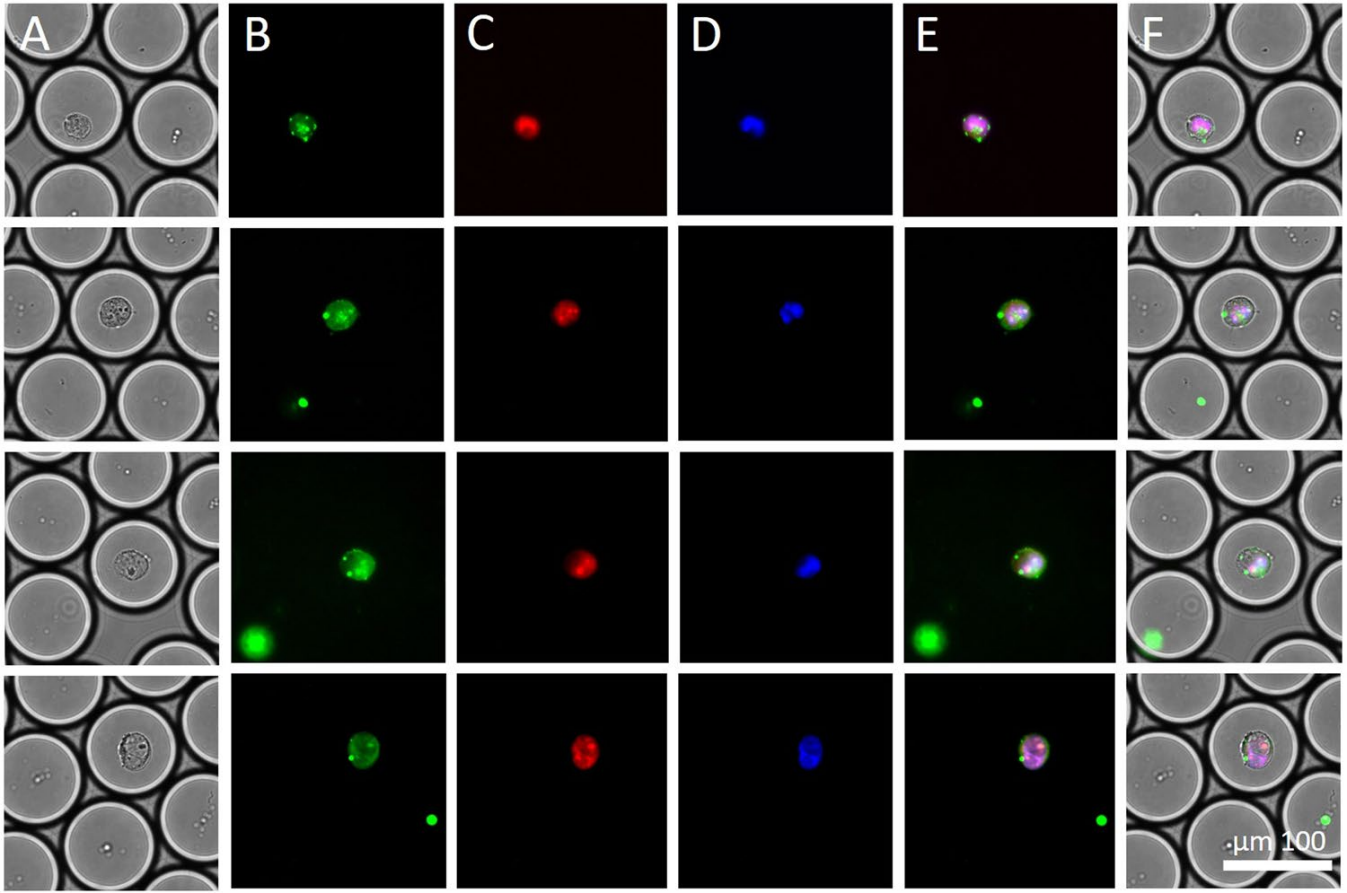
Overview of an electrofusion result. The electrofused HL60 cells inside the droplet contain two nuclei with different colors and a rearranged cell membrane. The droplet size is 50 pL. Six pulses of 1.2 kV/cm were applied. (**A**) Brightfield image of two electrofused HL60 cells in a droplet. (**B**) GFP image of two electrofused HL60 cells in a droplet. (**C**) RFP image of two electrofused HL60 cells in a droplet. (**D**) DAPI image of two electrofused HL60 cells in a droplet. (**E**) Overlay of DAPI, RFP GFP images of two electrofused HL60 cells in a droplet. (**F**) Overlay of brightfield, DAPI, RFP, and GFP images of two electrofused HL60 cells in a droplet.

The procedure to assess electrofusion was as follows. Simply the observation of two nuclei in a single cell would be insufficient to conclude electrofusion, since at the start of the electrofusion experiments, the HL60 cells were in the exponential growth phase, and thus the suspension also contained polynucleated cells (0.8%; 4/492). Electrofusion could therefore only be concluded if there were two differently and clearly distinctively labeled nuclei. To accurately assess cell electrofusion in a droplet, we therefore used different fluorescent (nuclear and membrane) dyes, which enabled us to monitor and analyze the process in detail. To visualize electrofusion, which is defined as two fused HL60 cells with one rearranged cell membrane containing two nuclei, half of the HL60 cells were stained with a green membrane dye and a blue nuclear dye, and the other half with the same green membrane dye but with a red nuclear dye. After the experiment, droplets containing cells with a rearranged green membrane encapsulating both a blue and a red nucleus were observed (Fig. 3). Several control experiments were performed. Before each electrofusion experiment, the two separately dyed cell populations were checked for successful staining under the microscope. Staining was found to be successful as, at the start of the experiment and in control experiments (no electric field applied), none of the cells contained a blue and a red nucleus with a green fluorescent membrane. Also, control experiments to check whether the cell dye transferred to other cells over time, were performed by removing the supernatant from the stained cells after thirty minutes and introducing it to a new cell population. Fluorescent images taken over time showed that no fluorescence was visible, ruling out cell dye transfer.

We found that six pulses of 0.71 ms at an estimated cell membrane voltage of about 450 mV resulted in electrofusion. The overall efficiency of the platform is 0.76%. In 5 different experiments (n = 5), on average 500 droplets were examined per experiment. In total 19 successful electrofusion events were found resulting in an overall efficiency of 0.76% (19/(5*500)). However, for a Poisson distribution with λ = 1, on average 15% of the droplets contain two cells. Thus, 15% of 500 droplets is 75 droplets, likely to contain two cells and qualify for possible electrofusion. Therefore the 19 successful fusion events lead to 5.06% (19/(5*75)) electrofusion efficiency.

## Discussion

In some of the droplets small string shaped particles are visible, which were not visible in freshly made and filtered electrofusion buffer. Further investigation also ruled out a yeast contamination, concluding that the string shaped particles were dust fragments. Other, more ellipse shaped particles are also visible in the droplets. After using filtered electrofusion buffer, the particles were still present in the droplets. Because some of these particles only have a green fluorescent signal we determined that these ellipse shaped particles are cell membrane fragments. Moreover, ellipse shaped particles without a fluorescent signal are possibly cell fragments escaped from leaky electroporated cells. The established efficiency is comparable to the efficiency of both commercially available methods^56–58^ and on-chip methods^55,59–61^. To better compete with these existing methods, there are several realistic opportunities to improve the electrofusion efficiency. First, the cell-cell contact can be enhanced geometrically (e.g. by placing a serpentine channel structure in front of the DC electric field to enhance cell movement^62^ or by implementing a droplet shrinkage module causing continuous cell-cell contact^32^) or electrically (e.g., by applying an AC electric field to improve cell alignment and cell-cell contact by DEP^63^). In addition, a 3D electrode configuration can be designed in such a way that it generates a homogeneous electric field along the channel wall, giving higher fusion efficiency.

We have shown single cell encapsulation efficiencies up to 77%^33^, droplet pairing and electrocoalescence up to 95%, and droplet shrinkage up to 75%^32^. By combining this platform with the platform demonstrated in previous work we expect to reach higher yields, because a higher percentage of droplets will be filled with two different cell types, and droplets will be smaller enabling better cell-cell contact^32,33^. Also the further coupling of this platform with picoinjectors to secure cell viability inside the droplets^64^, and a microfluidic platform capable of screening functional hybridomas^65^ is a possibility, and can be a major step towards functional hybridoma creation on chip. Finally, the use of the platform presented here is not restricted to cell fusion since it is applicable for various other cell-based assays such as single cell analysis and differentiation assays. Moreover, due to the membrane electroporation of the encapsulated cells, these cells are more likely to uptake desired components like small molecules and plasmid vectors^66^. In conclusion, we have shown electrofusion of cells in droplets, with exchange of nuclear components.

## Methods

### Chip design

The complete design consists of three functional components (Fig. 1A). Firstly, the cell solution is introduced in the yin-yang shaped inlet, that functions as an elongated funnel to gradually introduce the cells into the channel to prevent cells from clogging up the channel after which a component for encapsulation of cells in droplets (Fig. 1B), using a flow-focusing geometry. Secondly, a component for cell electrofusion in a droplet (Fig. 1C). Thirdly, a component for electrofusion: the droplets containing the cells pass six electrode pairs, which generate sufficient electric field strength in the droplets to induce electrofusion. The channel dimensions are 50 × 25 µm width × height (blue structures in Fig. 1A) and the electrode distance (yellow structures in Fig. 1A) and electrode width are 20 µm.

### Chip fabrication

The top part of the chip was made in polydimethylsiloxane (PDMS) (Sylgard 184, Dow Corning). Curing and base agent were mixed at a ratio of 1:10 and degassed. A silicon master design was drawn in Clewin (version 4.0.1) and fabricated using standard UV-lithography. SU-8 (Microchem) was spun on the silicon master with a thickness of 25 μm. PDMS was poured onto the silicon wafer, degassed, and cured at 60 °C for 24 hours. After curing, in- and outlets were punched using a dispensing tip

(Nordson EFD, inner Ø 1.36 mm and outer Ø 1.65 mm). The bottom part of the chip was fabricated by sputtering recessed platinum electrodes on a glass wafer using a standard sputtering technique and the glass wafer was diced into individual chips. The glass chip was bonded with UV-glue onto a printed circuit board (PCB). The electrode pads on both the PCB and the glass chip were connected via a double wirebond, which was sealed off with a drop of two component glue (Heisel). Onto the PCB, a plug was soldered, to make the electrical connection with the pulse generator (Agilent 33250 A). For electroporation and electrofusion in droplets, a DC voltage was applied, varying from 2 to 3 V.

Subsequently, the PDMS was bonded to the bottom part of the chip using an oxygen plasma (Harrick PDC-001). After bonding, the chip was placed at 60 °C for a minimum of 30 minutes. Before use, 10 mM FDTS in HFE7500 was introduced into the channels to ensure the hydrophobicity of the channel walls^67^.

### Cell membrane voltage model

To calculate a single applied electric field pulse over the cell membrane, an equivalent electrical circuit model was built as shown in Fig. 2E. The circuit consists of a number of coupled resistances and capacitances. The change in double layer capacitance of the second electrode during movement of the droplet with cells over the electrodes leads to a continuously changing charging of the capacitances in the system. It was assumed that the electrode directly contacts the electrofusion buffer, and that the capacitance of the electrofusion buffer/electrode interface was 20 μF/cm^2^. For the cell membranes the capacitance of a surface with a thickness of 5 nm and a relative permittivity of 2 was taken^51^. The conductance of the electrofusion buffer was 0.009 S/m and of the cell cytoplasm was 60 S/m and were used to calculate the resistances. The electrode distance and width were set at 25 µm and 20 µm respectively, similar to the dimensions in our microfluidic device. In our model the droplet encapsulated two HL60 cells with a diameter of 13 µm. The first electrode had a potential of 3 V, while the other electrode was set to ground potential (0 V). For the velocity of the droplet through the microchannel the measured value of 28 mm/s was used.

In our model t = 0 was taken at the moment that the droplet was fully in contact with the first electrode and started to move over the second electrode. All these parameters were included in the equivalent electrical circuit model as shown in Fig. 2E. The transient behavior of the circuit has been numerically approximated using a MATLAB script.

## Materials

HL60 (human promyelocytic leukemic) cells were grown in RPMI medium (Invitrogen), supplemented with 20% (v/v) fetal bovine serum (FBS; Invitrogen), 100 IU ml^−1^ penicillin (Invitrogen), 100 µg ml^−1^ streptomycin (Invitrogen) and 2 mM L-glutamine (Invitrogen) ( = RPMI + medium). Cell cultures were sustained in a 5% CO_2_ humidified atmosphere at 37 °C. Cell cultures were split every 3–4 days at a ratio of 1:10. Prior to the experiments, the cells were counted, to establish the correct volume fraction of 1.0% in electrofusion buffer (EF buffer). EF buffer is an isoosmolar low conducting buffer^22^, consisting of deionized water, supplemented with 280 mM inositol (Sigma), 0.1 mM calcium acetate (Sigma), 0.5 mM magnesium acetate (Sigma) and 1 mM l-histidine (Sigma). The conductivity was adjusted, using deionized water, to 0.009 S m^−1^. The cell containing solutions were used as the dispersed phase. For the continuous phase, HFE 7500 (3M^TM^ NOVEC^TM^) was used, with a 1% (wt/wt) oil-phase biocompatible fluorosurfactant. This surfactant was a kind donation of dr. Ralph Sperling (Harvard).

### Experimental set-up

The HL60 cell suspension and the oil suspension were introduced into the microfluidic chip using 1 mL plastic syringes (Braun) with a needle (BD Microlance). All syringes were connected to their matching inlets with TYGON tubing (inner Ø 0.02 inch and outer Ø 0.06 inch). Pipette tips were placed in the outlets of the chip and used as a waste container or as a collection vessel for the main outlet.

The flow was driven at a constant volume rate by a syringe pump (neMESYS dosing units). A magnetic stirrer bar was inserted into the cell containing syringes, to prevent the suspension from settling during the injection. The dispersed phase flow rates, containing the cell suspensions, were set at 0.2–0.25 µL min^−1^, the flow rate of the continuous phase at 1–2 µL min^−1^. The microfluidic chip was mounted onto the X–Y–Z translation stage of an inverted wide fluorescence microscope (Leica DM IRM, Leica Microsystems). A computer-controlled high-speed camera (Photron SA-3) was mounted onto the microscope for image recording, using the provided Photron software (Photron Fastcam Viewer). Illumination was supplied by a Leica lamp 12 V/100 W in combination with a condenser.

For the electrofusion experiments, various nuclear dyes (blue (NucBlue), yellow/orange [5 µM] (Cyto82), and membrane fluorescent dyes (green [5 µM] (Vybrant green), were used, as specified in the result section. All fluorescent stains were purchased from Invitrogen. Cells were stained according to the provided protocol. After the electroporation and electrofusion experiments, the droplets were collected and loaded on a microscope slide with single shallow depression (Corning). This allowed for the formation of a monolayer which could be scanned and analyzed.

### Image acquisition and analysis

Images and videos were captured using the high-speed camera, at a frame rate of 3000 frames per second and a shutter time of 50 µs. The captured images and videos were analyzed with the image-processing program ImageJ (National Institute of Health). Images of the fluorescently labeled cells in droplets were captured using a Life Technologies EVOS FL microscope with DAPI, RFP and GFP filter cubes.
